# Incorporating geographical factors with artificial neural networks to predict reference values of erythrocyte sedimentation rate

**DOI:** 10.1186/1476-072X-12-11

**Published:** 2013-03-12

**Authors:** Qingsheng Yang, Kevin M Mwenda, Miao Ge

**Affiliations:** 1 School of Resources and Environment, Guangdong University of Business Studies, Guangzhou, Guangdong, China; 2 Department of Geography, University of Lethbridge, Lethbridge, AB, Canada; 3 Department of Geography, University of California, Santa Barbara, CA, USA; 4 Department of Geography, Shaanxi Normal University, Shaanxi, China

**Keywords:** ESR, Geographical factors, Artificial Neural Network, Back propagation

## Abstract

**Background:**

The measurement of the Erythrocyte Sedimentation Rate (ESR) value is a standard procedure performed during a typical blood test. In order to formulate a unified standard of establishing reference ESR values, this paper presents a novel prediction model in which local normal ESR values and corresponding geographical factors are used to predict reference ESR values using multi-layer feed-forward artificial neural networks (ANN).

**Methods and findings:**

Local normal ESR values were obtained from hospital data, while geographical factors that include altitude, sunshine hours, relative humidity, temperature and precipitation were obtained from the National Geographical Data Information Centre in China.

The results show that predicted values are statistically in agreement with measured values. Model results exhibit significant agreement between training data and test data. Consequently, the model is used to predict the unseen local reference ESR values.

**Conclusions:**

Reference ESR values can be established with geographical factors by using artificial intelligence techniques. ANN is an effective method for simulating and predicting reference ESR values because of its ability to model nonlinear and complex relationships.

## Introduction

The erythrocyte sedimentation rate (ESR) is a well-established clinical test in diseased patients that is commonly used for estimating the body's acute phase reaction to inflammation and infection [1,2]. For many years, physicians have found normal ESR values useful for predicting specific disease severity and assessing general sickness index, among other uses. The origin of the concept of the ESR dates back to the early 19th century, when the Greeks observed the relation between the sedimentation of red blood cells and fibrinogen [3]. In 1918, Fahraeus discovered that erythrocyte sedimentation in plasma occurred more rapidly in pregnant women than they did in non-pregnant women [4]. Since then, with minor modifications, the ESR has been used in the evaluation of variety of diseases.

The most commonly used method of measuring the ESR is the Wintrobe method that is performed using a 100-mm tube containing oxalate as the main anticoagulant [5]. In order to compare the difference between the ESR value of patients and normal ESR value, the reference ESR values were measured in local hospitals and research institutes. Some studies have found that in addition to the reference ESR values varying with seasonal changes, they also have a significant variation with the age, gender, smoking habits and weight of patients [6-8]. One study proposed a formula for calculating the maximum normal ESR at any given age [8]. In the aforementioned study, the ESR value is calculated as (age in years/2) for men and (age in years +10)/2 for women [8]. While ESR values increase as people become older [6], the tendency of the ESR values to increase with age flattens out after age 60 [7,9]. Other observations regarding ESR values include the following: a general pattern of high ESR values in spring and autumn and low values in summer, and a significant increase of mean ESR values due to smoking and obesity [7]. In addition to age, gender, smoking habit and weight of patient, some studies have found that normal ESR values also vary with geographical factors [10-12]. For example, some studies found that ESR is significantly correlated with altitude, latitude, relative humidity, mean annual temperature and annual precipitation [10-12]. In our study, we maintain the inclusion of five geographical factors similar to a previous study [12]; however, we replace latitude with annual sunshine hours because of the effects of seasonal variation on ESR values suggested by a study in which high ESR values were observed in the spring and autumn while low values were observed in the summer [7]. The decrease of reference ESR values is significantly associated with increase in altitude and decrease of relative humidity, mean annual temperature and annual precipitation [10-12]. In order to find how such geographical factors affect the reference ESR values, some studies modeled the relationship using stepwise regression [10-12]. While geographical factors have been found to improve the prediction accuracy of local reference ESR values, the reasons as to why they do so are not definitive due to cross-correlation: for example, humidity, temperature and precipitation generally decrease as altitude increases, while annual sunshine hours are affected by seasonal variation of the other geographical factors at a specific altitude. Consequently, the relationship between reference ESR values and geographical factors is nonlinear and thus complicated in a manner that introduces limitations when a stepwise regression statistical model is used, given the cross-correlation of the independent variables [13].

In solving variable cross-correlations when calculating reference ESR values by incorporating geographical factors, this paper presents a new method of simulating and predicting local reference ESR values using artificial neural networks (ANN). This proposed method has a number of advantages over other methods. First, the training procedure is simple and convenient because the parameter values are obtained automatically by neural networks. Second, the method is efficient because it uses the well-developed procedure of back-propagation training [14,15], such that it is able to deal with complex interactions among variables. Finally, the use of a neural network means that the variables do not have to be independent of each other. All in all, the proposed model structure is more robust and stable compared with linear regression models.

## Methods and materials

### Artificial Neural Network

An Artificial Neural Network (ANN) is a nonlinear regression method that is inspired by the way biological nervous systems, such as the brain, process information. ANNs have been widely applied in many disciplines with a high degree of difficulty. Neural networks, with their remarkable ability to derive meaning from nonlinear data, can be used to extract patterns and detect trends that are too complex to be evaluated by simple regression techniques. A trained neural network can be thought of as an ‘expert’ in the category of information it has been given to analyze. This ‘expert’ can then be used to provide projections given new situations of interest.

The basic processing units in a neural network are the so-called neurons or nodes which are organized in several layers. All the neurons, except those in the input layer, perform two simple processing functions: collecting the activation of the neurons in the previous layer and generating activation as the input to the following layer. The neurons in the input layer only send signals to the next layer but process input data.

The functions for addressing the interactions between neurons are straightforward. With *i* denoting the equivalent of a sender neuron in the input layer and *j* denoting a receiver neuron in the next layer, the collection function is given as:

(2-1) $$\mathrm{ne}t_{j}=\sum_{i}W_{\mathrm{ij}}I_{i}$$

Where I_*i*_ is the signal from neuron *i* of the sender layer, net_*j*_ is the collection signal for receiver neuron *j* in the next layer and W_*ij*_ is the parameter or weight that sums up the signals from different input nodes. The receiver neuron in the next layer creates activation in response to the signal net_*j*_. The activation then becomes the input for its next layer. The activation is usually created in the form of a sigmoid or linear function.

The learning process of neural networks entails determining the adaptive weights which are used to address the strengths of network interconnection between associated neurons. The values of the weights are not set by the users but rather are determined by the network during training. One of the most popular training methods is a back-propagation learning algorithm which iteratively minimizes an error function over the network (calculated) outputs and desired outputs on the basis of a training data set [14,15]. An advantage of the back-propagation neural network is that the learning algorithm is not programmed into the network a priori [16]. The weights are initially set by a random process. The error, computed as the difference between calculated and desired activation for the output neuron, is propagated back through the network and used to adjust the weights. The process of adjusting the weights according to the errors is repeated over many iterations until the error rate is minimized or reaches an acceptable level. Once the optimized weights have been obtained from the training data set, the network is ready for prediction. Prediction is based on the activation level (*I*_*i*_) in the output neuron. The activation level of a neuron ranges from 0 to 1, a scale which reflects the variation from extremely low to extremely high strength of membership, respectively.

### ANN-based reference ESR values training and predicting model

Since normal ESR values have significant correlations with age and gender [7,10-12], normal ESR values of specific age and gender are selected for training data. In this study, the normal ESR values of men older than 60 years are selected. Normal ESR values of 148 cases (N) are obtained from local hospitals and medical research institutes in China using the Wintrobe method [10]. To correspond with each normal ESR value (*V*), there are a series of geographical factors (*S*_*1*_, *S*_*2*_, *S*_*3*_, *S*_*4*_, *S*_*5*_) that are selected for predicting local reference ESR values. They are as follows:

1) *S*_*1*_ - Altitude (m);

2) *S*_*2*_ - Annual sunshine hours (hours);

3) *S*_*3*_ - Annual average relative humidity (%);

4) *S*_*4*_ - Annual average temperature (°C);

5) *S*_*5*_ - Annual average precipitation (mm).

The Geographical data listed above are obtained from National Geographical Data Information Center China.

**Figure 1:**
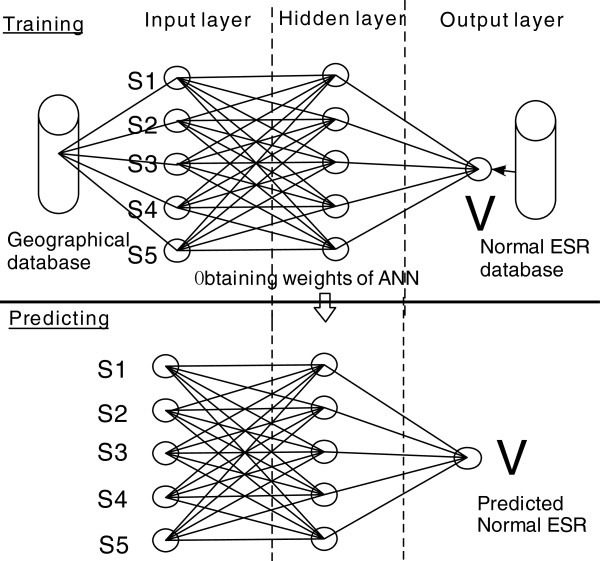
The ANN model for training and predicting reference ESR values.

A linear regression model may not be the best way to reveal relationships between ESR values and geographical factors because of the complexity inherent in the variables. Instead, we take advantage of neural networks in order to estimate normal ESR value at each city. Specifically, an ANN model is developed with *S*_*1*_, *S*_*2*_, *S*_*3*_, *S*_*4*_, and *S*_*5*_ as inputs and normal ESR values as desired output. The model consists of two separate steps: the training of the neural network to obtain the optimal weights automatically based on the input data, and the use of the neural network to predict local reference ESR values based on the trained ANN (Figure 1).

An essential task is to design the network structure for the prediction. The design of the network structure is simplified because the numbers of layers and neurons in the layers can be subjectively determined. However, an increase in the number of layers and neurons will drastically increase the computation time for the model. The principle is to use as few layers and neurons as possible without severely compromising model accuracy. Based on tests specific to our data, it is sufficient to use 3 layers in the neural network: one input layer, one hidden layer, and one output layer. The input layer has five neurons corresponding to the five geographical factors chosen for the study. There are 5 neurons in the hidden layer (Figure 1). The output layer has only one neuron which indicates normal ESR value. There are 25 (5×5) weights to be determined for the links between the input layer and the hidden layer, and 5 weights between the hidden layer and output layer. Consequently, a total of 30 parameters are used for the neural network model.

Experiments indicate that it would be more appropriate for all original data to be converted into a range from 0 to 1 before they are used in neural networks [17]. This approach is similar to data normalization in that it uses maximum and minimum values in scaling the original data set. Scaling variables ensures that they are all considered equally important inputs to neural networks and makes them compatible with the sigmoid activation function that produces a value between 0 and 1. The following linear transformation is used:

(2-2) $$S_{k}^{'}=\frac{S_{k}-S_{\min}}{S_{\max}-S_{\min}}$$

In this study, the signal for each neuron in the input layer is the normalized value of corresponding geographical factors:

(2-3) $$\mathrm{ne}t_{i}\left(x\right)=S_{k}^{'}\left(x\right)$$

Where *x* is a case, *net*_*i*_(*x*) is the received signal for neuron *i* of case *x* in the input layer, and *S*_*k*_^’^(*x*) is the *k*^th^ geographical factor of case *x.*

The transfer functions for the input layer and the first hidden layer are dictated by the *logsig* function. The *logsig* function is as follows:

(2-4) $$\frac{1}{1+\exp\left(-\mathrm{ne}t_{i}\left(x\right)\right)}$$

Based on the transfer function above, the signal received by neuron *j* of the hidden layer from neuron *i* of the input layer for case *x* is calculated as follows:

(2-5) $$\mathrm{ne}t_{1,j}\left(x\right)=\sum_{i}W_{\mathrm{ij}}\frac{1}{1+\exp\left(-\mathrm{ne}t_{i}\left(x\right)\right)}$$

Where *net*_1,*j*_(*x*) is the signal received by the first neuron *j* of the hidden layer for case *x, W*_*ij*_ is the weight from neuron *i* of the input layer to neuron *j* of the hidden layer.

The transfer function for the hidden layer and the output layer is the *purelin* function. The signal received by a neuron of the output layer from neuron *i* of the hidden layer for case *x* is calculated as follows:

(2-6) $$O\left(x\right)=\sum_{i}W_{i}\mathrm{ne}t_{1,i}\left(x\right)$$

Where *O*(*x*) is the signal received by a neuron of the output layer for case *x, W*_*i*_ is the weight from neuron *i* of the hidden layer to a neuron of the output layer.

The network was trained using the *Levenberg-Marquardt* (LM) algorithm. The values of these weights are automatically determined by the learning process which is based on the back-propagation algorithm in the MATLAB Neural Network toolbox.

The whole set of samples is automatically divided into three groups of 70%, 15% and 15% for the learning process of neural networks. The first group is the training dataset and the others are validation dataset and test dataset, respectively. The training dataset is used to obtain the weights for each link between a pair of neurons, the validation dataset is used to generalize the training data and the test dataset is then used to verify the learning results. A set of weights is finally obtained from the training process. One of the most important characteristics of trained neural networks is their ability to generalize training data from validation data. If the network simply memorizes or overfits the training data, it will generally perform poorly on the test data. It is important to decide the number of iterations so that the training can be stopped properly. If the training process takes too long, the network may be overtrained and consequently cause large prediction errors for the test data. In this study, the aforementioned potential problem was averted by stopping the training when the error of the validation data began to increase.

## Results and discussion

The structure of the neural network is shown in Table 1.

**Table 1:** The structure and specification of ANN

**Neural network**	**Multi-layer perception**
No. of hidden layers	1
No. of neurons in the input layer	5
No. of neurons in the hidden layer	5
No. of neurons in the output layer	1
Learning rate	0.5
No. of epochs	28
Adaption learning function	Levenberg-Marquardt (LM) algorithm
Training error	4.4875

The error difference (ΔV) and error deviation (ΔV %) for the normal ESR value (V) are evaluated as:

(3-1) $$\mathrm{\Delta V}=V_{\mathrm{meas}}-V_{\mathrm{pred}}$$

(3-2) $$\mathrm{\Delta V\%}=100*\frac{V_{\mathrm{meas}}-V_{\mathrm{pred}}}{V_{\mathrm{meas}}}$$

Where *meas* and *pred* stand for measured and predicted values respectively.

The Average Absolute Deviation (AAD%) is evaluated as:

(3-3) $$\mathrm{AAD\%}=\frac{1}{N}\sum_{i=1}^{n}\left|\mathrm{\Delta V\%}\right|$$

Where N is the number of cases.

The maximum and minimum of ΔV along with AAD % are shown in Table 2.

**Table 2:** The maximum and minimum of ΔV and AAD %

**Data**	**Train**	**Test**
Maximum ΔV	6.91	6.61
Minimum ΔV	0.0036	0.0758
AAD %	0.12	0.143

**Figure 2:**
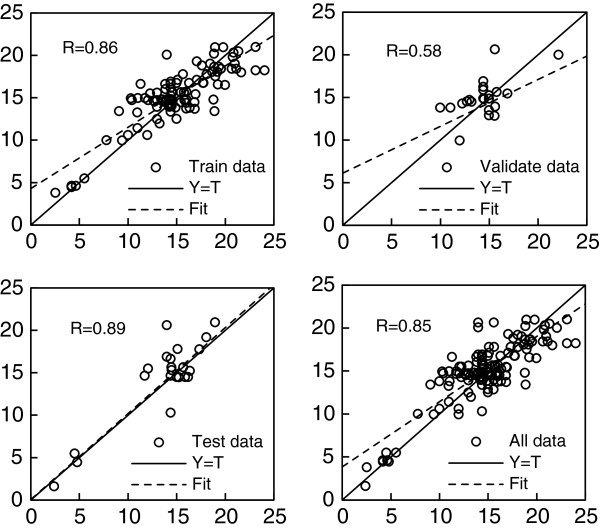
Comparison of ANN regression among Training data, Validation data, Test data and all data.

Based on the proposed ANN model, it is observed from Table 2 that the maximum deviation for the training data set is less than 6.91% and the maximum deviation for the test data set is less than 6.61%. This indicates that there is a good agreement between trained and predicted data. The comparison of normal ESR value (V_meas_.) with predicted normal ESR value (V _pred_.), error difference and error deviation for some of training and test data are shown in Table 3. The results show that the predicted values are in statistically in agreement with measured data. The comparison of ANN regression among training data, validation data, test data and all data are respectively shown in Figure 2. The regression coefficient of test data and all data is higher than training data, which indicates that the trained ANN model is reliable as well. These figures also show the predicted values are very close to measured values with minimal error.

**Table 3:** **Comparison of the measured ESR (V**_**meas**_**) with predicted ESR (V**_**pred**_**), error difference, error deviation**

**ANN input**					**ANN output**	**V**_**meas **_**(mm/h)**	**Error**	
**Altitude (m)**	**Annual sunshine hours (hours)**	**Annual average relative humidity (%)**	**Annual average temperature (°C)**	**Annual average precipitation (mm)**	**V**_**pred **_**(mm/h)**		**|ΔV|**	**ΔV%**
6.3	1906.0	79.0	21.8	1694.1	18.03	18.68	0.65	0.03
30.0	2200.0	78.0	15.5	1070.0	20.18	18.86	1.32	0.07
31.2	2780.2	60.0	11.5	644.2	16.73	17.78	1.05	0.06
38.0	2600.0	69.0	13.9	701.8	15.70	16	0.30	0.02
41.6	2574.0	65.0	7.8	734.5	14.69	12.07	2.62	0.22
46.7	1903.9	77.0	17.5	1596.4	14.64	12.66	1.98	0.16
50.0	2737.3	58.0	14.2	685.0	13.78	16.36	2.58	0.16
62.8	2700.0	69.0	12.5	700.0	16.01	13.87	2.14	0.15
63.2	2100.0	81.0	20.8	1400.0	19.77	20.77	1.00	0.05
72.2	1827.0	79.0	21.6	1300.6	13.38	18.9	5.52	0.29
75.9	2173.0	74.0	15.1	1109.0	20.04	14	6.04	0.43
80.0	2737.8	62.0	12.9	549.9	16.61	14.4	2.21	0.15
84.0	1848.2	77.0	19.6	1343.7	14.80	14.4	0.40	0.03
97.0	2700.0	65.0	10.1	656.0	19.01	21	1.99	0.09
100.6	1680.0	78.0	18.0	1300.0	14.71	10.4	4.31	0.41
110.4	2385.3	66.0	14.2	640.4	16.14	14.4	1.74	0.12
147.0	2867.0	62.0	3.2	415.5	13.91	14.3	0.39	0.03
150.0	2200.0	68.0	3.9	700.0	13.95	14.2	0.25	0.02
166.7	1600.0	79.0	19.1	1926.0	13.25	10.97	2.28	0.21
171.7	2641.0	67.0	3.6	523.3	14.47	16.86	2.39	0.14
200.0	3053.0	59.0	5.8	376.0	14.37	16	1.63	0.10
241.4	2600.0	69.0	3.9	550.0	14.55	13.9	0.65	0.05
260.6	1100.0	78.0	18.0	1130.0	14.74	13.46	1.28	0.10
723.9	2900.0	58.0	7.7	406.0	14.93	11.75	3.18	0.27
777.9	2675.8	60.0	9.5	459.5	13.81	14.89	1.08	0.07
843.9	1250.0	80.0	15.2	1098.0	12.46	13	0.54	0.04
1050.0	2970.5	55.0	5.8	417.5	12.49	15	2.51	0.17
1071.2	1371.0	79.0	15.3	1174.7	11.94	13.25	1.31	0.10
1111.5	3039.6	59.0	8.5	202.8	9.93	9.42	0.51	0.05
1517.2	2607.6	59.0	9.1	327.7	10.57	12	1.43	0.12
2275.0	2762.0	56.0	5.9	343.1	5.46	5.54	0.08	0.01
2780.0	3210.0	32.0	4.3	38.8	11.39	11	0.39	0.04
3658.0	3007.7	45.0	7.5	454.0	4.58	4.21	0.37	0.09
3800.0	3000.0	38.0	−0.7	60.0	3.75	2.55	1.20	0.47
4080.0	2350.0	62.0	−3.2	510.0	4.43	4.2	0.23	0.05

Since the trained ANN is reliable, reference ESR values can be predicted using the trained ANN model. For example, the reference ESR values of the cities of Lhasa, Guiyang, Yinchuan, Nanchang and Beijing are predicted as shown in Table 4.

**Table 4:** Predicted reference ESR values

**City**	**Geographical factors**					**ANN output**
	**Altitude (m)**	**Annual sunshine hours (hours)**	**Annual average relative humidity (%)**	**Annual average temperature (°C)**	**Annual average precipitation (mm)**	**V**_**pred **_**(mm/h)**
Lhasa	3658.0	3007.7	45	7.5	454.0	4.58
Guiyang	1071.2	1371.0	79	15.3	1174.7	11.94
Yinchuan	1111.5	3039.6	59	8.5	202.8	9.93
Nanchang	46.7	1903.9	77	17.5	1596.4	14.64
Beijing	31.2	2780.2	60	11.5	644.2	16.73

The original data, result data and ANN model parameters can be downloaded from additional file 1.

It is important to explore the complex relationship between local ESR values and geographical factors. Studies have shown that the ESR values decrease with increase in altitude because oxygen content gradually decreases while altitude rises [10-12]. As a result, the amount of red blood cells increases, inducing a fall in ESR reference value in healthy subjects [11,18]. The decrease in ESR reference value is correlated with a decrease in temperature, humidity and precipitation, all of which affect annual sunshine hours based on seasonal variation. Therefore, while incorporating all the aforementioned geographical factors can help explain ESR differences within similar altitudes, the relationship is nonlinear and thus complicated, hence the use of ANN to model the dependent relationships.

## Conclusions

In this study, a multi-layer feed-forward neural network model has been developed to predict the local reference ESR values, taking into account corresponding local geographical factors. The network is trained using training data, after which the latter is then used to predict unseen reference ESR values. The results show that predicted values are in statistical agreement to measured values. The use of ANN is an effective method for simulating and predicting reference ESR values because of its ability to model nonlinear and complex relationships. The main advantage of our proposed method is the simplicity and stability of the model structure. Although the reference ESR values can be predicted with geographical factors by using artificial neural networks, the reason why reference ESR values vary with geographical factors should be further explored.

## Competing interests

The authors declare that they have no competing interests.

## Authors' contributions

QSY carried out the design of the study and performed the statistical analyses. KM conceived of the study, and helped to revise the manuscript. MG acquired and interpreted the data. All authors have read and approved the final manuscript.

## Supplementary Material

Additional file 1: **Original data, result data and ANN model parameters.** (RAR 47 kb) Click here for file
